# Segmentation of Melanocytic Lesion Images Using Gamma Correction with Clustering of Keypoint Descriptors

**DOI:** 10.3390/diagnostics11081366

**Published:** 2021-07-29

**Authors:** Damilola Okuboyejo, Oludayo O. Olugbara

**Affiliations:** ICT and Society Research Group, South Africa Luban Workshop, Durban University of Technology, Durban 4000, South Africa; dokuboyejo@ieee.org

**Keywords:** data clustering, dermoscopy image, gamma correction, image segmentation, keypoint descriptor, melanocytic lesion

## Abstract

The early detection of skin cancer, especially through the examination of lesions with malignant characteristics, has been reported to significantly decrease the potential fatalities. Segmentation of the regions that contain the actual lesions is one of the most widely used steps for achieving an automated diagnostic process of skin lesions. However, accurate segmentation of skin lesions has proven to be a challenging task in medical imaging because of the intrinsic factors such as the existence of undesirable artifacts and the complexity surrounding the seamless acquisition of lesion images. In this paper, we have introduced a novel algorithm based on gamma correction with clustering of keypoint descriptors for accurate segmentation of lesion areas in dermoscopy images. The algorithm was tested on dermoscopy images acquired from the publicly available dataset of Pedro Hispano hospital to achieve compelling equidistant sensitivity, specificity, and accuracy scores of 87.29%, 99.54%, and 96.02%, respectively. Moreover, the validation of the algorithm on a subset of heavily noised skin lesion images collected from the public dataset of International Skin Imaging Collaboration has yielded the equidistant sensitivity, specificity, and accuracy scores of 80.59%, 100.00%, and 94.98%, respectively. The performance results are propitious when compared to those obtained with existing modern algorithms using the same standard benchmark datasets and performance evaluation indices.

## 1. Introduction

### 1.1. Background

Melanocytic lesions typically refer to the proliferation of melanin-producing neural crest-derived melanocytic cells in human skin. These lesions can either be benign (innocuous) or malignant (cancerous). The top three prevalent malignant melanocytic lesions include basal cell carcinoma (BCC), squamous cell carcinoma (SCC), and melanoma [1]. Melanoma is a skin cancer that is often caused by an unpredictable disorder in the melanocytic cells, which eventually leads to the improper synthesis of melanin. It is currently the 7th most commonly occurring cancer in young adults within the age bracket of 15–29 [1]. Moreover, it is one of the most contributing factors toward the disability-adjusted life years (DALYs) of many countries within North America, West Europe, and Australia [2]. The socio-economic impacts for patients with melanoma have increased in certain geographical areas. A recent report has indicated a relationship between unemployment and impaired health-related quality of life (HRQoL) in melanoma patients in a geographical region of China [3].

The need for early detection of skin cancer cannot be overemphasized because a 5-year survival rate has been reported to be up to 95% for melanoma diagnosed at an early stage [4,5]. Dermatologists mostly rely on excision biopsy of lesion areas and subject the extracted lesions to clinical examination to have a successful early detection. Concerns with frequent lesion excision include blisters, infections, bleeding, and sometimes nerve damage. Literature has indicated a low confidence interval of less than 40% when excision is performed to detect cancerous lesions [6]. This inherent challenge has necessitated the drive to espouse non-invasive and automated diagnosis for classifying a lesion as benign or malignant. Segmentation is a crucial preprocessing step to successfully distinguish between benign and malignant lesions to reduce avoidable excisions that can be economically costly both emotionally and mentally [1,7]. However, several challenges are facing the accurate segmentation of skin lesions. They include the presence of noise artifacts, low contrast surrounding skin, fuzzy borders, and irregular structures that characterize lesion images. A good number of dermatologists rely heavily on manual tumor tracing for lesion localization. This is, however, prone to human error, which gives room for inconsistencies [1,7]. Several lesion segmentation methods have been proposed in the literature to address the concerns for aiding better lesion diagnosis.

The extraction of a set of promising features that can help to localize lesion areas is considered an essential component to effectively perform lesion boundary tracing. A feature can be described as a piece of information relevant to solving a specific problem of data analytics. In addition, features can be used to reference interesting points in an image. The interesting points would typically have well-defined positions and are often invariant to affine transformations such as scale, rotation, and illumination. These features can be based on low-level image primitives, such as intensity (color), shape (edge, region, contour), and texture (co-occurrence matrix, Fourier). The connotation of keypoint features generally refers to spatial locations that define interesting points in an image. These interesting points can be of varying characteristics, such as edge, corner, line, and blob. The use of keypoint properties can be useful for detecting lesion areas. The extraction of appropriate discriminating features that can aid effective lesion segmentation is often influenced by conditions surrounding the acquisition of lesion images. Such conditions include non-uniform illumination that can affect image contrasts. This is one application domain where preprocessing techniques, such as gamma correction, can be applied faithfully. The method of gamma correction, which is sometimes called gamma adjustment, can be applied to lesion images to obtain the desired contrast features for effective lesion boundary tracing.

The common challenges often associated with many of the existing image segmentation methods include the difficulty of method reproducibility, computational intensiveness, and performance bottleneck. The purpose of this study is to introduce a simple, reproducible, and effective novel method based on gamma correction with clustering of keypoint descriptors to improve the segmentation performance of melanocytic lesions. The performance of the proposed lesion segmentation algorithm has been compared with a set of modern methods on two standard publicly available benchmark datasets using the popular evaluation metrics. This study has contributed to the research on skin lesion segmentation through the development of a novel segmentation algorithm with the following unique feats:

- The application of keypoint descriptors with a data clustering technique for accurate segmentation of skin lesion areas, which recorded an improved segmentation performed when compared to the existing algorithms for the same task.
- The transformation of the identified keypoint descriptors by the clustering of valid neighboring image data points to identify the true lesion area of interest.
- The experimental comparison of the proposed image segmentation algorithm with other prominent image segmentation algorithms reported in the literature to demonstrate the effectiveness of the proposed algorithm. The remainder of this paper is succinctly organized as follows. Section 1.2 highlights the related works while Section 2 describes the introduced method. Section 3 discusses the experimental results, and Section 4 gives a concluding remark.

### 1.2. Related Works

The literature on image processing and computer vision has reported advances in the use of several lesion segmentation approaches, such as edge-based [8], region-based [7,9], contour-based [10], texture-aware [11], thresholding [12,13], clustering [14,15], and, recently, deep learning [16,17,18]. The edge-based image segmentation methods typically rely on edge operators such as Laplacian of Gaussian (LOG) and Canny to retrieve relevant edge information that can assist in boundary tracing. However, edge-based image segmentation customarily suffers from the use of dynamic programming on lesions comprising several tumor areas [1]. Region-based segmentation algorithms mostly group lesion areas using collective image characteristics [19,20]. However, it might sometimes lead to over-segmentation, especially for lesion images comprising of multi-colored areas [1]. The contour-based lesion segmentation provides the ability to either use region or edge information to estimate lesion boundaries [19,20]. Texture-based segmentation relies on textual properties, such as co-occurrence matrices of lesion images, to suggest possible lesion boundaries. Thresholding techniques can be useful for lesion segmentation by assigning pixels below or above a threshold value. However, one major logjam to the use of thresholding is its unpredictability when applied to images with noise such as vignettes. Most methods of segmentation by clustering follow the pattern of thresholding in multidimensional space to trace lesion boundaries in an unsupervised manner. The study reported in [13] utilized an amalgam of methods comprising histogram intensity equalization, thresholding, morphological operation, and a GrabCut algorithm to segment lesion areas in dermoscopic images. The thresholding technique was used for the first-level segmentation in the CIELAB color model, while Grabcut was used to perform the second-stage semi-automatic segmentation.

In recent years, there has been traction in the application of deep learning methods for the segmentation of skin lesions. Deep learning algorithms are well suited for semantic segmentation of images, pixel-wise labeling, and automatic feature construction, which have all yielded remarkable performances [21,22,23,24,25,26,27,28,29]. Deep learning methods for skin lesion segmentation reported in the literature are mostly based on the convolutional neural network (CNN) because of its inherent ability to leverage large datasets to extract discriminative object features. The CNN is a special type of deep neural network (DNN) where each neuron in the network layers has multiple high-dimensional filters that are convolved with the input of the current or previous layer. Liu et al. [23] proposed a deep learning method based on CNN with auxiliary information to segment skin lesions, achieving up to 88.76% sensitivity. Similarly, Phan et al. [26] utilized two auxiliary tasks integrated into a decoder of U-Net architecture for skin lesion segmentation to report accuracy up to 94.55%. Khan et al. [30] implemented a hybrid approach that uses maximum mutual information to fuse results from a CNN model and an improved high-dimension contrast transform (HDCT)-based saliency segmentation. They proposed a 16-layered CNN architecture that consisted of five convolutional layers, four rectified linear activation function (ReLu) layers, three max-pooling layers, one transpose layer, one SoftMax, and a one-pixel classification layer. The authors concluded that they extracted more discriminant features using the localized lesion regions when compared to extraction of features from the original images. However, one of the main challenges of using deep learning methods is their tendency to have overfitted models.

The application of keypoints to segment regions of interest in images has gained momentum in recent years. Lin et al. [31] applied keypoint contexts to detect region duplication within an image. Heinly et al. [32] reported the performance of popular feature detectors and descriptors for perspective, affine image, and non-geometric transforms. The strength of keypoint-based segmentation lies in the effectiveness of the identified keypoint features and possibly with corresponding descriptors. Feature detection is the process of identifying interesting points within a given image that can uniquely characterize an image. Several detectors of keypoint features have been reported in the literature, including good features to track (GFTT) [33], Harris detector [34], level curve curvation, features from accelerated segment test (FAST) [35], adaptive and generic accelerated segment test (AGAST) [36], maximally stable extremal regions (MSER), Laplacian-of-Gaussian (LoG), difference-of-Gaussian (DoG), and determinant-of-Hessian (DoH). In the context of lesion images, a feature detector refers to an algorithm that can detect interest points within a given image. Single-scale feature detectors, such as the Harris detector and FAST detectors, have a single representation for the detected features. Multi-scale feature detectors, such as LoG and DoG, have multiple representations. Patch feature description refers to a squared pixel that represents a neighborhood around a given interest point.

Feature description is the process of representing the characteristics of a given set of image features, and it refers to an algorithm that can be used to represent unique image characteristics. It typically encodes feature points into a series of numbers that can be used as a numerical fingerprint to distinguish one feature from another. Some of the frequently used descriptors include binary robust independent elementary features (BRIEF) [37,38] and fast retina keypoint (FREAK) [39]. In addition, the literature has recorded several algorithms with the capability to both detect and describe keypoints. In this category are speeded-up robust features (SURF) [40,41], scale-invariant feature transform (SIFT) [42], oriented FAST and rotated BRIEF (ORB) [43], binary robust invariant scalable keypoints (BRISK) [44], KAZE [45], and accelerated KAZE (AKAZE).

Previous studies have compared the effectiveness of various feature descriptors and their strength in terms of invariant to scale, rotation, and viewpoint changes [46,47,48,49,50]. However, the success could sometimes be soiled if applied to poorly acquired images and medium-heavy noised lesion images. Due to undesired results emanating from poorly acquired images, there has been a growing need to have some sort of correction that can easily compensate for this intrinsic curb. The use of gamma is one correction to influence the intensity of a given image when performing the required morphological operation. Baptiste et al. [51] proposed a new edge detector based on anisotropic linear filtering, local maximization, and gamma correction. Their method has boasted the ability to detect edges in parts of a given image where objects are either under-exposed or over-exposed. The application of gamma correction with keypoint descriptors is introduced to effectively segment lesions in dermoscopy images.

## 2. Materials and Methods

### 2.1. Materials

Publicly available experimental datasets from two prominent sources were used to test the performance of the proposed segmentation algorithm. All the 200 images, consisting of 160 benign Melanocytic Nevus (MN) with 40 malignant Melanoma (MM) from the Dermatology service of Pedro Hispano hospital (PH2) dataset, were used for comparison of the proposed algorithm with existing non-deep learning algorithms. In addition, 5400 images of 4014 Melanocytic Nevi (MN), 399 Seborrheic Keratosis (SK), 19 Actinic Keratosis (AK), 50 Dermatofibroma (DF), 40 Vascular Lesion (VL), 13 Unknown Benign Types (UNB), 657 Melanoma (MM), 101 Basal Cell Carcinoma (BCC), 44 Squamous Cell Carcinoma (SCM), and 63 Unknown Malignant Types (UNM) selected from the international skin imaging collaboration (ISIC) dataset were used for comparison. Table 1 illustrates the description of the experimental images used to test the performance of the proposed algorithm for the segmentation of skin lesions. The images used for experimentation are characterized by moderate to heavy noise, such as air bubbles, hair occlusion, ruler marking, and vignette. It should be noted that these images were featured in ISIC challenges in the years 2016 to 2019.

### 2.2. Proposed Method

A novel method termed gamma-adjusted skin image segmentation using keypoint (GASISUK) is offered to precisely segment skin lesion areas from melanocytic images. The GASISUK algorithm is invariant to scale, orientation, and rotation transformations. The technique of gamma correction is used to obtain the desired contrast features for a given lesion image. The essential keypoints are then extracted from the gamma-corrected image as discriminating features relative to lesion areas and surrounding non-lesion areas. The extracted keypoints are then clustered in a way that can foster an appropriate and inexpensive segmentation of lesion areas. The publicly available implementations of source codes of modern non-deep learning methods were used to evaluate the segmentation results. The code implementations used for both efficient graph-based image segmentation [52] and statistical region merging [53] were provided in [54]. In this study, to evaluate the saliency detection method [55], we relied on the application provided in [56]. The source code implementations for the rest of the other non-deep learning methods were from the study reported in [57]. The essential phases of the proposed algorithm are image preprocessing, gamma correction, and clustering of keypoint features.

#### 2.2.1. Image Preprocessing

Image preprocessing was performed on a given image I with a domain of definition D to increase the efficiency of the lesion segmentation algorithm because of the possible presence of noise in I that might negatively affect the segmentation result. The edge-preserving image smoothing function proposed by Ambrosio and Tortorelli [58] was applied to reduce the effect of unwanted artifacts such as salt and pepper noise particles in the lesion image. Ambrosio and Tortorelli [58] have validated in their work that active contour based on the Mumford–Shah functional given by Equation (1) can be derived by computing the limit of energy functional $E\left|J,\;B,\;\varepsilon \right|$ where boundary B is replaced by continuous function ⱬ, whose magnitude indicates the presence of a boundary. The image smoothening operation can equally assist in highlighting the possible hair occlusion while blurring the image background. This was particularly beneficial in the identification of hair shaft noise from lesion images.

(1) $$E\left|J,\;B,\;\varepsilon \right|=C\int {\left(I\left(\vec{x}\right)-J\left(\vec{x}\right)\right)}^{2}d\vec{x}+A\int ⱬ\left(\vec{x}\right){\left|\vec{\nabla }J\left(\vec{x}\right)\right|}^{2}d\vec{x}+B\int \left\{\varepsilon {\left|{\vec{\nabla }}_{ⱬ}\left(\vec{x}\right)\right|}^{2}+\varepsilon^{-1}\varphi^{2}\left(ⱬ\left(\vec{x}\right)\right)\right\}d\vec{x}$$

where $\varphi \left(ⱬ\right)$ is a potential function with the following possible solutions:

(2) $$\varphi_{1}\left(ⱬ\right)=\frac{1-ⱬ}{2};\;ⱬ\in \left|0,1\right|$$

(3) $$\varphi_{2}\left(ⱬ\right)=3ⱬ\left(1-ⱬ\right);\;ⱬ\in \left|0,1\right|$$

A fast line detector (FLD) [59] was used to detect hair shaft noise in the given input lesion image, and the threshold length of detectable lines restricted to a size of 20 has improved performance results in this study. FLD is a recognition algorithm that uses a vocabulary tree built with mean standard deviation line descriptors to find candidate matches. The morphology black-hat operation was then applied on the results of the FLD to further eliminate possible noise, such as hair shaft and ruler marking, from the lesion image. Different structuring kernel sizes were used depending on the size of the detected lines. The actual removal of the identified lines was performed using a digital inpainting method of the fast marching method (FMM) [60,61]. This method ensures that a lesion image is free from noise such as hair shaft and ruler marking that often confuses most segmentation methods. Algorithm 1 summarizes the essential steps performed on each lesion image during the preprocessing stage to achieve effective segmentation results.
**Algorithm 1.** Preprocessing.$I_{a}$ = smoothening of image $I_{s}$ using Ambrosio-Tortorelli [58] minimizer $I_{g}$ = gray level of $I_{a}$ $L_{f}$ = fast line detections using length threshold of 20 Len$\left(L_{f}\right)$$=\mathrm{size}\;\mathrm{of}\;\mathrm{detected}\;L_{f}$ if Len$\left(L_{f}\right)\geq 1$  create a 2-d kernel $k_{bm}$ based on the Len$\left(L_{f}\right)$     $k_{bm}=3\times 3$ If 20 ≥ Len$\left(L_{f}\right)$ ≥ 1     $k_{bm}=7\times 7$ 35 ≥ Len$\left(L_{f}\right)$ 20     $k_{bm}=11\times 11$ If 50 ≥ Len$\left(L_{f}\right)$ 35     $k_{bm}=15\times 15$ If Len$\left(L_{f}\right)$ 50  $I_{bm}$ = morphology blackhat of $I_{g}$ using $k_{bm}$  $I_{bt}$ = morphology binary threshold of $I_{bm}$  $I_{p}$ = fast marching inpaint [61] of $I_{bt}$ else  $I_{p}$ = initialize as $I_{a}$

#### 2.2.2. Gamma Correction

The application of gamma correction to lesion images can help to ensure that low contrast images are properly adjusted to reduce the effect of local shadow and suppress noise interference on the images. Moreover, it can help to optimize the usage of image bits by taking advantage of humans’ non-linear perception of light and color. Due to the complexity surrounding the acquisition of lesion images, digital images can have undesirable quality. Gamma correction has been applied in this study to obtain the desired contrast features for a given image and assist in enhancing lesion thresholding [62,63].

#### 2.2.3. Clustering of Keypoint Descriptors

Image features can be categorized appositely as flat, edges, corners, or blobs in computer vision. Flat areas within an image refer to regions where pixel intensities tend to be homogeneous. Edges refer to boundaries between regions of an image where there is a discontinuity in pixel values. Corner features represent points in an image where two edges intersect and often reveal regions where there is a maximum intensity. It reveals the regions where there is the maximum variation in intensity when moved in all directions within an image. Blobs refer to dark on bright regions or bright on dark regions within an image. When a window is moved vertically on an image, a flat region yields the same result, but edges and corners might produce different results. Similarly, when a window is moved horizontally on an image, both the flat and edge regions produce repeatable results parallel to the direction. However, corner regions would probably produce an irreproducible result when such a window is moved horizontally, making it valuable for identifying discriminating features within an image. Consequently, the GASISUK algorithm uses corner keypoints as discriminating features relative to the lesion areas and surrounding non-lesion areas. In addition, it uses ORB [43] to perform keypoint feature detection and description because ORB descriptors can detect keypoints at each pyramid level, giving it a scale invariance advantage. Moreover, it advances the success of BRIEF that uses binary strings to represent feature points by adding rotation invariant capability at a much computationally cheaper rate. Furthermore, feature detectors in ORB leverage the achievement of the FAST feature detector using a multiscale representation of a single image at different resolutions, thereby adding scale-invariant capability. Moreover, it adds the orientation capability to the FAST algorithm to successfully detect interest points at a much quicker speed. An orientation is assigned to each keypoint depending on the level of intensity change around each keypoint.

The image pixels of lesion areas were observed to be typically situated in closed proximity regions. This suggests that features representing lesion areas could likely form well-defined density-connected components that can aid in appropriate lesion boundary tracing. Density-based spatial clustering of applications with noise (DBSCAN) is a non-parametric clustering algorithm based on pixel density [64,65]. It marks a point as a cluster outlier if it lies in a low-density region where its nearest neighbors are far apart. The clustering algorithm is particularly advantageous over partition-based counterparts, such as the k-means algorithm or the Fuzzy c-means algorithm, because of its ability to find arbitrarily shaped clusters without requiring a user to specify the number of clusters a priori. The application of DBSCAN can help to ensure that noise particles masquerading as features are trapped as outliers. In this study, we have clustered the identified keypoint features from lesion images using the DBSCAN algorithm with Euclidean distance metric in a memory-efficient way. In addition, we have ensured that groups with less than two contiguous members are automatically discarded from the list as potential noise. The density-connectedness and density-reachability mechanisms were computed as detailed in the previous study [64].

### 2.3. Algorithmic Description

Algorithm 2 gives the description of GASISUK being proposed in this paper to effectively segment lesion areas of interest from the surrounding regions. The brightness of each lesion was classified as predominantly light or dark relative to the dominant color of a given lesion image, and the dominant color was computed using a flattened array bin count. The algorithm showcases two-level contour filtering based on well-defined conditions. Due to the application of morphology operations, such as erosion in the first-level contour filtering, we have realized that the surface area of the identified lesion boundary could potentially reduce. This can influence the condition of the second-level contour filtering to ensure that the identified contours have a surface area relative to a pre-defined minimum value.
**Algorithm 2.** GASISUK Algorithm.Let $I_{p}$ = preprocessed image (see Section 2.2.1) Let $o_{w}$ and $o_{h}$ be the original width and height of $I_{p}$ respectively Let č = dominant color of $I_{p}$ If $č>\left(20,\;20,\;20\right)$  $B_{r}$ = predominantly light else  $B_{r}$ = predominantly dark if $B_{r}$ is predominantly light  assume gamma factor $g_{f}$ of 0.75  $I_{\gamma }$ = gamma of $I_{p}$  $I_{\gamma_{g}}$ = gray of $I_{\gamma }$  $I_{\gamma_{t}}$ = binary threshold of $I_{\gamma_{g}}$  $I_{\gamma_{m}}$ = morphology opening of $I_{\gamma_{t}}$ using a 7 × 7 matrix  $I_{\gamma_{b}}$ = initial segmentation using bitwise_AND of $I_{\gamma_{m}}$ and $I_{p}$  If $I_{\gamma_{b}}$ == [0] … depicting black image    $I_{\gamma_{b}}$ = $I_{p}$ else  assume gamma factor $g_{f}$ of 1.7  $I_{\gamma_{b}}$ = gamma of $I_{p}$ $k_{p}$ = keypoint features of $I_{\gamma_{b}}$ using ORB $k_{p_{c}}$ = clustering of keypoint features using DBSCAN $C_{i}$ = contour sketch of clustered keypoints $I_{\gamma_{f_{1}}}$ = first-level contour filtering  filter-off contours satisfying below characteristics
    ∗ contour is identified as inner contour    ∗ contour has an area of less than 32.000    ∗ width and height of contour less than minimum width (variable, value of $0.2o_{w}$ or fixed value of 200) and minimum height (variable value of $0.2o_{h}$ or fixed value of 200)  morphology erosion of contours $I_{\gamma_{f_{2}}}$ = second-level contour filtering  filter-off contours satisfying below characteristics
    ∗ ccontour has an area of less than 25.000
$I_{s}$ = segmented lesion image using filled mask of $I_{\gamma_{f_{2}}}$


## 3. Results and Discussion

This section presents the experimental evaluation of the GASISUK algorithm on the PH2 database and subsets of the ISIC database containing lesion images with heavy noise, such as vignettes, hair follicles, ruler marking, and air bubbles. The qualitative and quantitative test results of the algorithm against modern segmentation algorithms are also presented and discussed in this section.

### 3.1. Qualitative Result

Table 2 shows the qualitative results computed by GASISUK against some of the modern non-deep learning algorithms. Out of the 5400 images in the ISIC dataset and 200 images in the PH2 dataset used in this study, we have selected 60 image subsets comprising 15 benign ISIC images, 15 benign PH2 images, 15 malignant ISIC images, and 15 malignant PH2 images for qualitative evaluation. The modern algorithms evaluated include morphology active contour without edge (morph_cv_ls) [10], morphology geodesic active contour (morph_gac_ls) [10], saliency detection (saliency_map) [55], simple linear iterative clustering (slic_clust) [66], and statistical region merging (srm_obj) [53]. The proposed method of this study shows compelling visual results and increased prowess over the comparative methods.

Most of the existing modern algorithms failed to properly trace the lesion boundary if occluded with artifacts such as hair or ruler marking, as seen in the sample comparison of ISIC_0000043, ISIC_0000095, and PH2_IMD003. As detailed in Section 2.2.1, our algorithm resolved many of the challenging noise artifact images by applying preprocessing operations, such as Ambrosio and Tortorelli [58], fast line detector (FLD) [59], the fast marching method (FMM) [60], and morphological operations such as Blackhat. Some of the lesion images, such as SIC_0000004, ISIC_0000030, ISIC_0000147, ISIC_0000179, ISIC_0000554, IISIC_0001108, ISIC_0001118, ISIC_0001142, PH2_IMD168, PH2_IMD349, and PH2_IMD435, exhibit multiple shades of intensity that could easily be confused as lesion areas in the segmentation procedure. The vignette noise was another artifact that was seen to have negatively influenced the outcome of most of the evaluated algorithms, as seen in Table 2 for ISIC_0000125, ISIC_0000247, and ISIC_0000249, Table 3 for PH2_IMD010, PH2_IMD048, and PH2_IMD375, Table 4 for ISIC_0000004, ISIC_0000030, and ISIC_0001142, and Table 5 for PH2_IMD064 and PH2_IMD348. However, in our proposed algorithm, the application of gamma correction on each of the lesion images has assisted in obtaining the desired contrast effect for a given lesion image. This mechanism has contributed greatly towards the superior outcome of our proposed algorithm, as detailed in Table 2, Table 3, Table 4 and Table 5, on either benign or malignant lesions using both PH2 images and heavily noised ISIC images.

### 3.2. Quantitative Result

The standard statistical evaluation metrics recommended in the literature were used to quantitatively assess the results computed by the proposed algorithm. For the geometric evaluation, we have used the median (Med) value for each of the following metrics: Med-sensitivity, Med-specificity, Med-accuracy, Med-Jaccard-index, and Med-Dice-coefficient. This is particularly beneficial given the resilience of equidistant values to outliers and ease of computation. Sensitivity measures the degree of correctly identified lesion areas. Specificity measures the degree of correctly identified non-lesion areas. The Jaccard index compares similarity and diversity between predicted lesion areas and actual ground truth. The Dice coefficient compares the pixel-wise similarity between the predicted lesion areas and actual ground truth. Accuracy measures the statistical bias of the lesion segmentation.

The proposed algorithm has been compared against several algorithms from the literature and we observed the median value to have performed with remarkable results. The algorithms used for the purpose of quantitative evaluation are active contour without edge (ACWE), tagged cv_ls [67], morphology ACWE (morph_cv_ls) [10], morphology geodesic active contour (Morphology GAC), tagged morph_gac_ls [10], adaptive thresholding (adaptive_thresh) [68], ISODATA thresholding (isodata_thresh) [69], mean thresholding (mean_thresh) [70], triangle thresholding (triangle_thresh) [71], Otsu thresholding (otsu_thresh) [72], saliency detection (saliency_map) [55], statistical region merging (srm_obj) [53], efficient graph-based image segmentation (egbs_obj) [52], and simple linear iterative clustering (slic_clust) [66]. The results with a Jaccard index ≥0.6 and Dice coefficient ≥0.6 are considered acceptable generally in literature, and therefore used as a benchmark in this study. In Table 6, adaptive thresholding (adaptive_thresh) [68] and Morphology GAC [10] recorded the best results, with sensitivity scores of 97.95% and 93.66%, respectively, over the benign ISIC dataset, reflecting the possibility of capturing most of the lesion interest points from the surrounding skin area. Considering the result of adaptive_thresh across other metrics, it can be observed to have performed poorly, as the median Jaccard index and Dice coefficient are both below 0.6. The proposed GASISUK method and morphology ACWE (morph_cv_ls) [10] recorded the best specificity score of 100% over the same dataset, which is then followed by statistical region merging (srm_obj) [53] with a specificity score of 99.96%. The competing specificity result of morph_cv_ls can be attributed to the usage of curvature morphological operators by the authors. The GASISUK algorithm has recorded a better result in accuracy (95.17%), Jaccard index (0.80), and Dice coefficient (0.89), showing its superiority when compared to the other algorithms.

The evaluation on benign PH2 images in Table 7 shows srm_obj [53] to have recorded the best sensitivity score, followed by Morphology GAC (morph_gac_ls) [10]. The proposed algorithm equally recorded superior results in accuracy (96.76%), Jaccard index (0.85), and Dice coefficient (0.92). In Table 8 and Table 9, Morphology ACWE (morph_cv_ls) [10] shows the best specificity result, while adaptive thresholding (adaptive_thresh) [68] shows promising results based on sensitivity score.

In Table 8, the proposed algorithm has displayed its superiority over the other algorithms when considering the accuracy (93.45%), Jaccard index (0.78), and Dice coefficient (0.88) results on the malignant ISIC dataset. Similar results were recorded for the proposed algorithm in Table 9 with accuracy (80.94%), Jaccard index (0.70), and Dice coefficient (0.82).

The srm_obj [53], as illustrated in Table 9, has performed the best with a sensitivity score of 99.66% over the malignant PH2 dataset, which is then trailed by adaptive thresholding (adaptive_thresh) [68]. Similar to the result obtained over the malignant ISIC dataset, Morphology ACWE [10] has recorded the best specificity score of 100% over the malignant PH2 dataset, which is then closely trailed by the proposed algorithm with a value of 99.50%. However, the accuracy (80.94%), Jaccard index (0.70), and Dice coefficient (0.82) showcase the strength of the proposed algorithm as topping the performance chart.

The explosion of deep learning methods for segmentation of lesion images has reported commendable results in recent years. In Table 10, some of the recent modern deep learning algorithms over the ISIC 2017 segmentation task have been highlighted. The authors of the deep learning works have reported performance results over 600 images from the ISIC datasets. Phan et al. [25] recorded the best accuracy, Jaccard index, and Dice coefficient. The accuracy recorded by Phan et al. [25] could be attributed to the two auxiliary tasks of boundary distance map regression and corresponding contour detection. The result by Shan et al. [27] recorded the highest specificity because it applied a dual-path network as a replacement for fully convolved DenseNets. The combined evaluation of our algorithm over 5400 ISIC images of benign and malignant, however, showed a promising result of 100.00% specificity, 94.98% accuracy, a 0.79 Jaccard index, and a 0.89 Dice coefficient, thus displaying favorable skin lesion generalization.

### 3.3. Discussion

Due to variation in the degree of noise per image, data preprocessing was discovered to improve the segmentation result of most of the test images. Noise artifacts such as hair follicles and ruler marking typically affect the segmentation results, thus necessitating the need to do some sort of initial removal of such artifacts. A fast line detector [59] with a line threshold of 20 was used to estimate the number of possible hair follicles and ruler markings represented as lines within a lesion image. Blackhat morphological operation was used to assist in estimating the traces of hair follicles or similar noise. The lengths of the detected lines were used to determine the kernel matrix for effective computation of the Blackhat operation. The mask generated from the Blackhat operation was then used to perform fast marching inpainting using the neighboring pixels. The Blackhat and inpainting operations were restricted to avoid unnecessary preprocessing of lesion images that have at least one detected line.

The keypoint feature detection process using the ORB detector was limited to 3000 to reduce the possibilities of noise masquerading as valid features. The ORB parameters were tuned to have a scale factor for pyramid decimation of 1.2, and the number of pyramid levels was limited to 8. The patch size and edge threshold for the oriented BRIEF descriptors were both computed as 9. The minimum acceptable contour area was set to 32,000 and 25,000 for the first-level and second-level contour filtering, respectively. To ensure noise artifacts are filtered off during first-level contour filtering, any contour less than 0.2 w × 0.2 h depicting 20% of the original width and 20% of the original height was discarded. The dimension assumption automatically falls back to a fixed minimum of 200 × 200 if the initial minimum required dimension fails to yield the acceptable segmentation result. Image intensity dominance was computed using 2D-array bin-count and RGB color range of (256, 256, 256). If the computed dominant color is greater than (20, 20, 20), the image is labeled as predominantly light, otherwise, it is labeled as a dark image. The brightness label further determines how the gamma correction of the image is performed. For light-labeled images, Otsu binary thresholding is performed, and if it does not yield the desired result, the procedure automatically falls back to the adaptive Gaussian binary thresholding. This is subsequently followed by gamma correction of the image intensity for the desired contrast. The usage of the ORB feature detector and descriptor in the proposed method ensures that the detected keypoints are invariant to basic transformations such as rotation, scale, and orientation. The DBSCAN of the identified keypoints was performed to ensure the detected keypoints that do not form part of the lesion areas are filtered. The clustering algorithm was used to filter groups with less than two contiguous members.

In this study, we tested our algorithm on the entire 200 images of the PH2 dataset and 5400 moderately to heavily noised lesion images from the ISIC dataset. The testing of our algorithm on the PH2 benign lesion images has yielded equidistant results of 89.42% sensitivity, 99.55% specificity, 96.76% accuracy, a 0.85 Jaccard index, and a 0.92 Dice coefficient. Compelling results of over 90% of the tested benign lesion images were seen to have a minimum of a 0.6 Jaccard index. Equidistant results of 70.39% sensitivity, 99.50% specificity, 80.94% accuracy, 0.70 Jaccard index, and 0.82 Dice coefficient scores were recorded over the selected malignant PH2 lesion images. The percentage of the malignant lesion images having a minimum of 0.6 Jaccard index score was seen to be 72.50%. Consequently, this has yielded an overall equidistant result of 87.29% sensitivity, 99.54% specificity, 96.02% accuracy, a 0.83 Jaccard index, and a 0.91 Dice coefficient over the entire 200 images from the PH2 dataset. Similarly, a convincing equidistant result of specificity, accuracy, Jaccard index, and Dice coefficient of 100.00%, 95.17%, 0.80, and 0.89, respectively, was recorded after testing our algorithm on the 4535 benign lesion images from the ISIC dataset. An improved result of 99.63% of the total benign lesion images from the ISIC dataset was seen to have at least a 0.6 Jaccard index. For malignant lesion images tested from the ISIC dataset, our algorithm achieved 80.02% sensitivity, 99.97% specificity, 93.45% accuracy, a 0.78 Jaccard index, and a 0.88 Dice coefficient. Up to 94.22% of the tested malignant lesion images from the ISIC dataset were seen to have a minimum of a 0.6 Jaccard index. Overall, our algorithm has yielded an enthralling result of 80.59% sensitivity, 100.00% specificity, 94.98% accuracy, 0.79 Jaccard index, and 0.89 Dice coefficient scores over the 5400 lesion images selected from the ISIC dataset.

**Figure 1:**
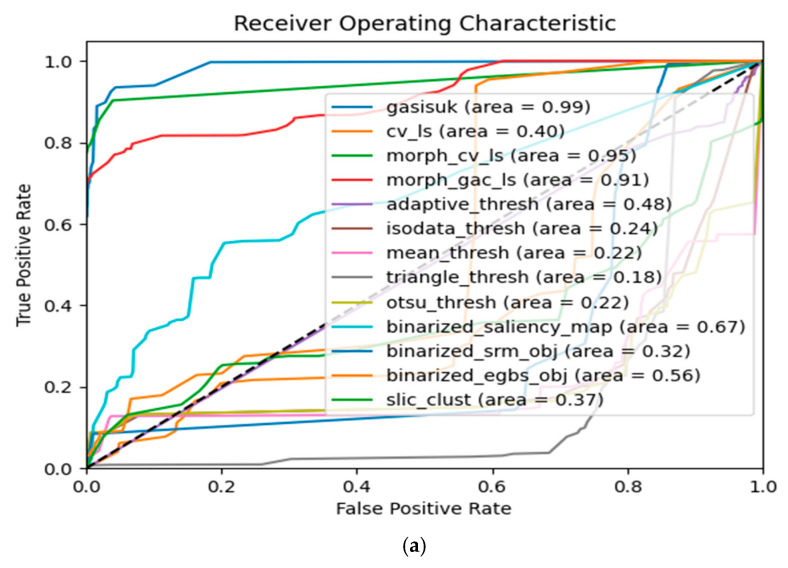

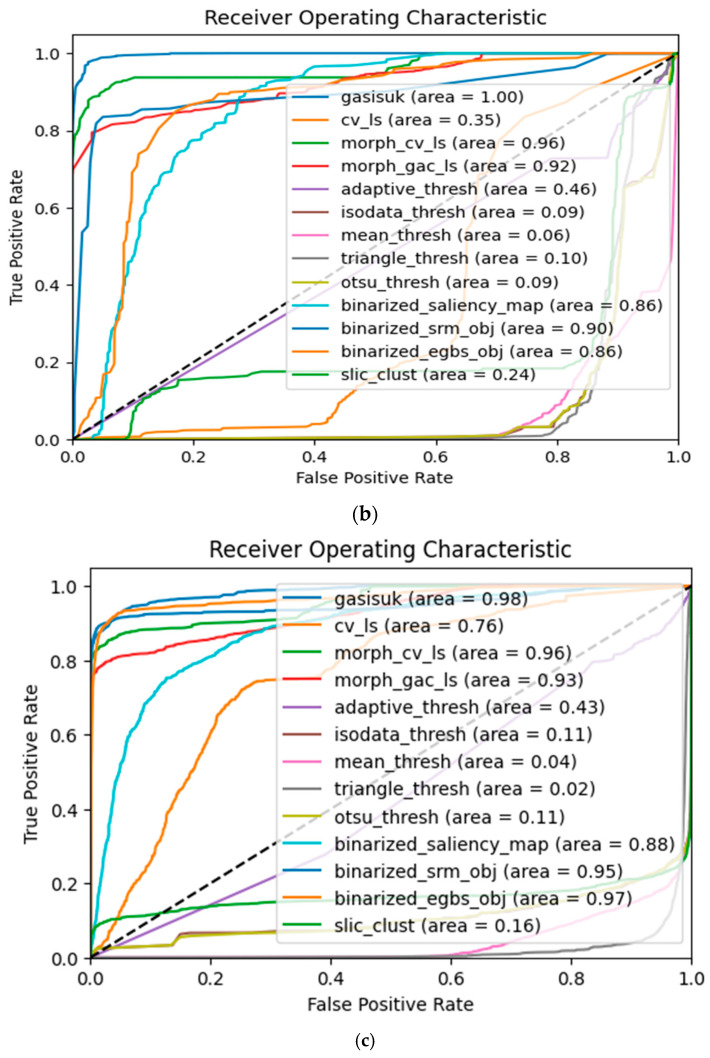

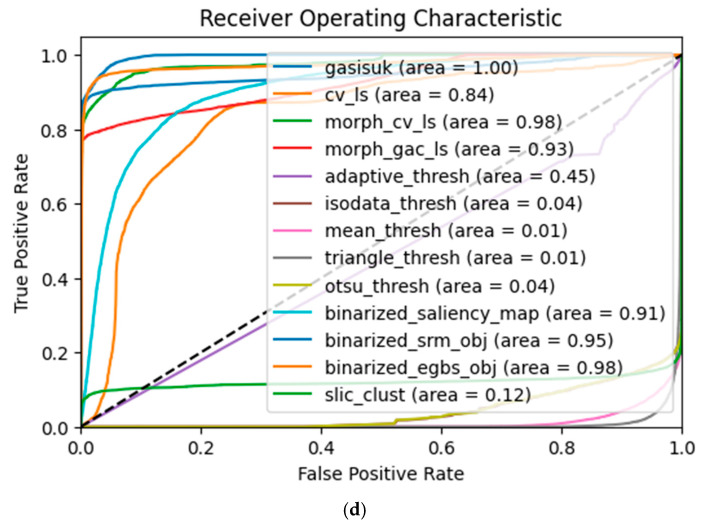
(**a**) ROC over PH2 Malignant; (**b**) ROC over PH2 Benign; (**c**) ROC over ISIC Malignant; (**d**) ROC over ISIC Benign.

The receiver operating characteristic (ROC) curve in Figure 1a–d illustrates the potential and effectiveness of our proposed algorithm. It has achieved a compelling area under the ROC curve (AUC) of 1.00 on benign lesion images from both PH2 and ISIC datasets. Similarly, an AUC result of 0.99 was respectively recorded for PH2 malignant and 0.98 for ISIC malignant lesion images. As illustrated in [74], we have considered an AUC value of a minimum of 0.70 acceptable and an AUC between 0.80 and 0.90 as excellent, while results over 0.90 are considered outstanding. The top-performing algorithms with a minimum AUC of 0.90 on both PH2 and ISIC datasets were Morphology ACWE, Morphology GAC, and the proposed GASISUK algorithm. Both Otsu and mean thresholding algorithms performed the worst on PH2 malignant lesion images, with an approximated value of 0.22 in the AUC result. Similarly, both Otsu and triangle thresholding reported the least-performing results for PH2 benign lesion images. The ISODATA thresholding, mean thresholding, triangle thresholding, Otsu thresholding, and simple linear iterative clustering performed the least on both ISIC malignant and benign lesion images. Statistical region merging (SRM) has performed well on both PH2 and ISIC benign lesion images, with AUC values of 0.90 and 0.95, respectively, though it has performed poorly when evaluated over malignant PH2 datasets. The efficient graph-based image segmentation (EGBS) appeared to be able to trace lesion boundaries of most ISIC datasets for both malignant and benign, reporting AUC values of 0.97 and 0.98 for malignant and benign lesion images, respectively. It was observed that EGBS has performed excellently on benign PH2 images, but poorly on malignant PH2 lesion images, like the behavior seen in SRM. The saliency detection method gave good results on benign ISIC lesion images and performed excellently on malignant ISIC lesion images.

Considering the 5400 datasets used for evaluation experimentation, we believe our method shows generalization in relation to skin lesion segmentation. It should also be noted that while deep learning methods have shown good promise in object classification challenges because of their learning ability using feature sets, recent literature reports have suggested that their accuracies in the domain of medical image segmentation need further improvement [75]. Deep learning segmentation methods have been reported to also lack pixel-level accuracy without the application of further processing [76,77]. This is primarily because most of them work on the feature level rather than the pixel level for image segmentation. In addition, the use of deep learning methods for image segmentation is currently being impaired because of factors such as the need for more datasets for continuous training, lack of memory-efficient models for both training and inference evaluation [76], limited reference information for accurate validation [78], and the possibility of over-fitted results [79].

## 4. Conclusions

The application of gamma correction, keypoint descriptors, and data clustering has assisted in the effective segmentation of melanocytic lesion images. The scaling of images to a standard dimension of 200 × 150 during the processing task has contributed towards increasing the execution speed of the proposed algorithm. Once the segmentation process is complete, the image is then rescaled to the desired dimension without loss of information. The novel application of gamma correction, keypoint features, and data clustering has been demonstrated for the segmentation of melanocytic lesion images. While it is important to identify multiple lesion areas within a given image, the proposed algorithm is highly effective. It can filter potential inner lesion areas if the outer areas have already been selected to avoid duplicate segmentation of the same lesion areas. This mechanism ensures that the proposed algorithm can effectively perform multiple segmentation of lesion areas without duplication of the segmented regions, such as segmented inner regions found within another segmentation region. The proposed lesion segmentation algorithm has proven to be compelling when compared to some modern segmentation algorithms, and it would be interesting to see how it contributes to an effective diagnosis of lesion images in clinical settings.

## Figures and Tables

**Table 1:** Experimental datasets.

Database	Benign						Malignant				Total
	MN	SK	AK	DF	VL	UNB	MM	BCC	SCC	UNM	
PH2	160						40				200
ISIC	4014	399	19	50	40	13	657	101	44	63	5400

**Table 2:** Qualitative evaluation on a subset of ISIC dataset (benign).

ID	Original Image	Ground Truth	GASISUK	MORPH_ CV_LS [10]	MORPH_ GAC_LS [10]	SALIENCY_ MAP [55]	SLIC_ CLUST [66]	SRM_OBJ [53]
**ISIC_0000042**	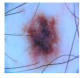	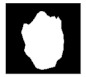	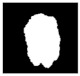	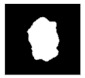	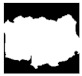	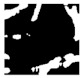	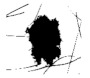	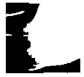
**ISIC_0000090**	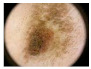	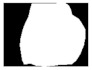	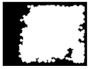	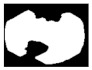	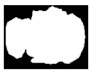	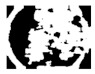	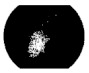	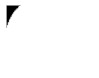
**ISIC_0000095**	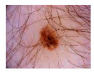	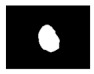	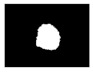	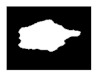	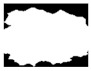	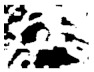	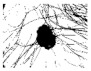	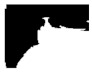
**ISIC_0000125**	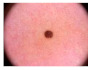	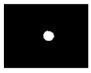	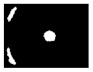	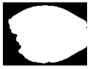	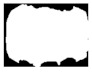	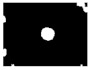	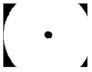	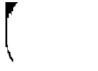
**ISIC_0000138**	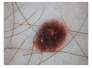	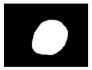	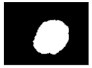	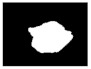	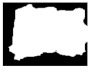	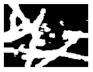	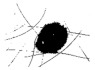	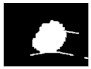
**ISIC_0000179**	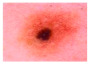	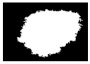	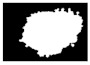	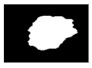	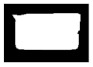	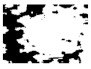	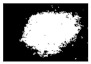	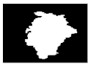
**ISIC_0000189**	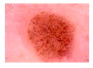	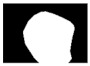	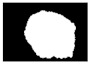	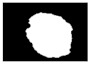	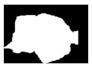	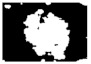	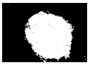	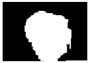
**ISIC_0000214**	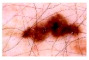	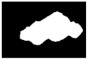	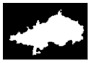	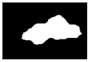	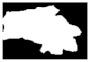	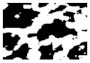	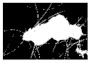	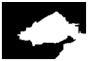
**ISIC_0000219**	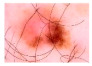	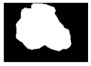	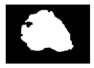	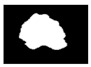	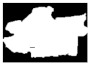	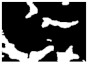	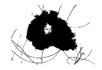	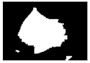
**ISIC_0000223**	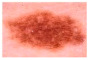	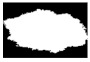	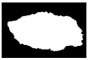	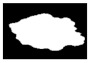	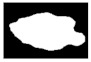	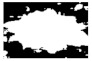	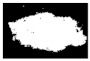	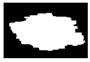
**ISIC_0000229**	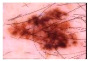	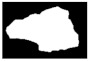	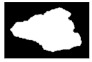	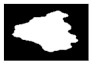	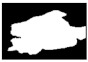	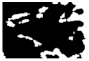	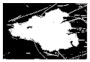	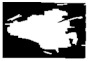
**ISIC_0000247**	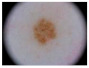	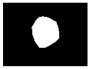	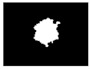	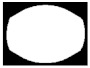	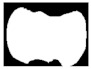	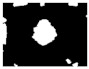	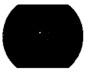	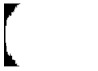
**ISIC_0000249**	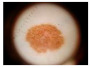	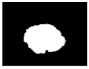	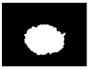	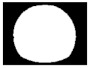	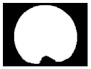	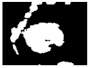	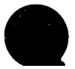	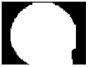
**ISIC_0000250**	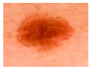	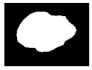	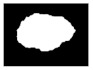	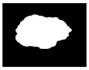	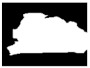	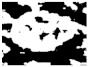	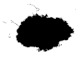	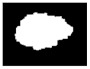
**ISIC_0000258**	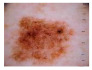	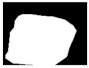	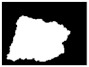	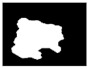	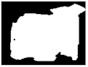	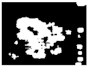	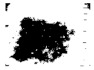	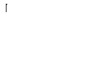

**Table 3:** Qualitative evaluation on a subset of PH2 dataset (benign).

ID	Original Image	Ground Truth	GASISUK	MORPH_CV_LS [10]	MORPH_GAC_LS [10]	SALIENCY_MAP [55]	SLIC_CLUST [66]	SRM_OBJ [53]
**PH2_IMD002**	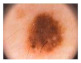	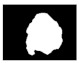	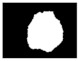	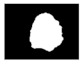	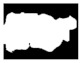	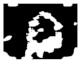	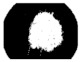	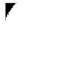
**PH2_IMD003**	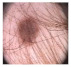	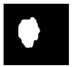	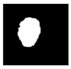	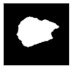	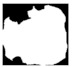	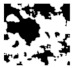	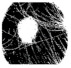	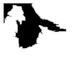
**PH2_IMD010**	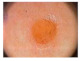	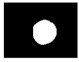	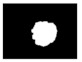	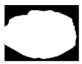	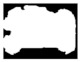	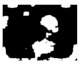	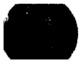	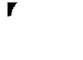
**PH2_IMD015**	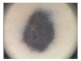	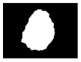	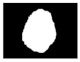	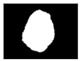	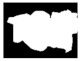	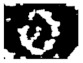	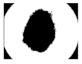	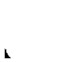
**PH2_IMD019**	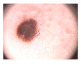	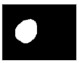	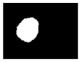	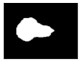	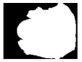	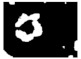	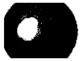	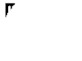
**PH2_IMD048**	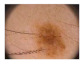	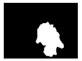	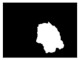	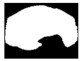	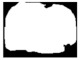	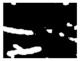	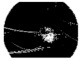	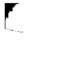
**PH2_IMD049**	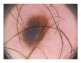	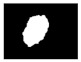	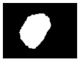	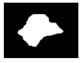	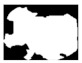	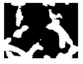	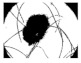	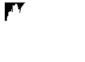
**PH2_IMD101**	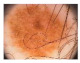	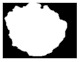	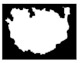	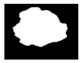	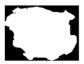	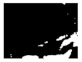	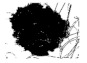	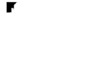
**PH2_IMD166**	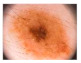	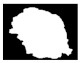	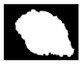	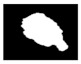	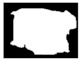	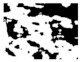	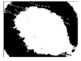	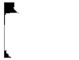
**PH2_IMD304**	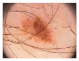	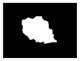	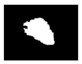	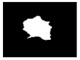	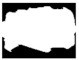	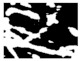	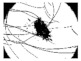	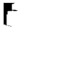
**PH2_IMD305**	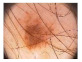	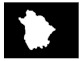	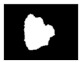	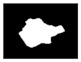	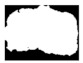	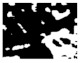	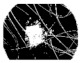	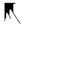
**PH2_IMD306**	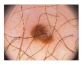	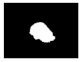	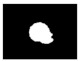	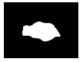	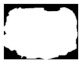	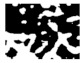	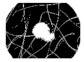	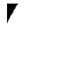
**PH2_IMD372**	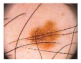	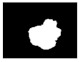	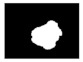	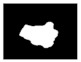	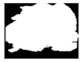	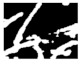	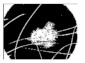	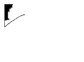
**PH2_IMD375**	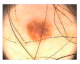	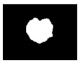	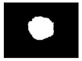	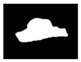	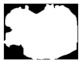	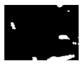	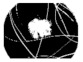	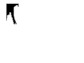
**PH2_IMD393**	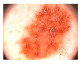	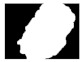	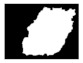	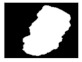	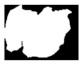	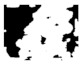	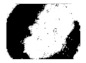	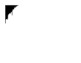

**Table 4:** Qualitative evaluation on a subset of ISIC dataset (malignant).

ID	Original Image	Ground Truth	GASISUK	MORPH_CV_LS [10]	MORPH_GAC_LS [10]	SALIENCY_MAP [55]	SLIC_CLUST [66]	SRM_OBJ [53]
**ISIC_0000004**	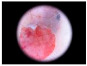	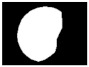	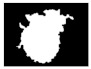	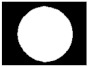	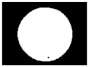	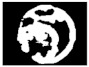	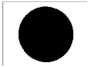	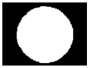
**ISIC_0000030**	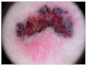	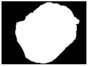	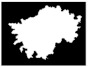	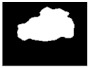	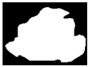	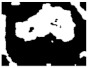	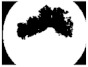	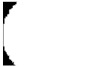
**ISIC_0000042**	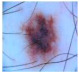	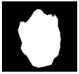	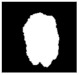	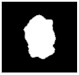	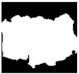	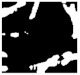	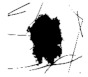	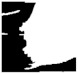
**ISIC_0000043**	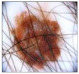	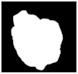	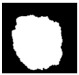	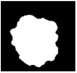	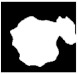	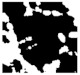	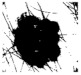	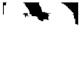
**ISIC_0000147**	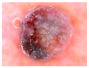	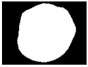	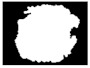	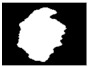	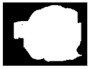	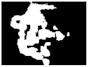	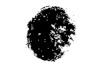	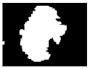
**ISIC_0000547**	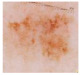	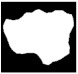	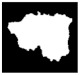	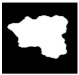	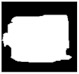	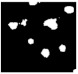	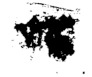	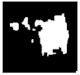
**ISIC_0000555**	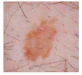	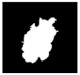	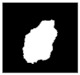	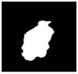	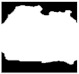	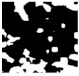	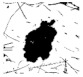	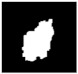
**ISIC_0001100**	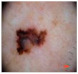	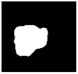	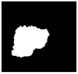	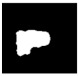	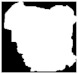	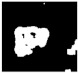	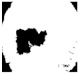	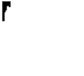
**ISIC_0001108**	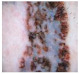	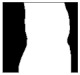	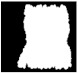	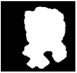	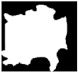	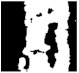	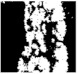	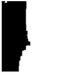
**ISIC_0001118**	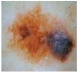	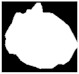	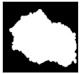	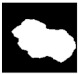	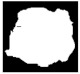	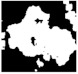	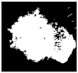	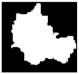
**ISIC_0001119**	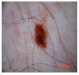	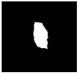	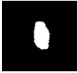	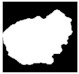	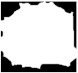	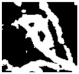	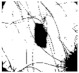	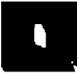
**ISIC_0001124**	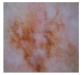	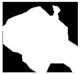	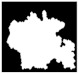	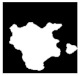	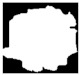	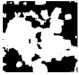	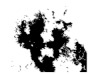	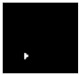
**ISIC_0001142**	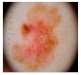	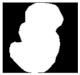	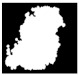	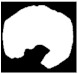	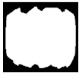	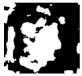	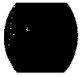	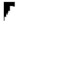

**Table 5:** Qualitative evaluation on a subset of PH2 dataset (malignant).

ID	Original Image	Ground Truth	GASISUK	MORPH_CV_LS [10]	MORPH_GAC_LS [10]	SALIENCY_MAP [55]	SLIC_CLUST [66]	SRM_OBJ [53]
**PH2_IMD063**	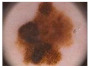	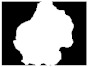	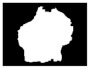	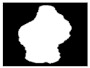	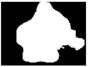	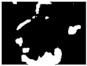	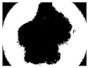	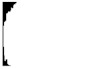
**PH2_IMD064**	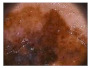	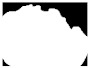	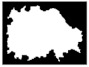	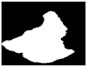	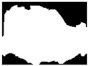	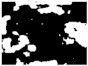	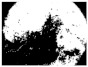	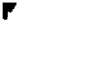
**PH2_IMD168**	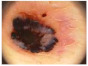	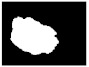	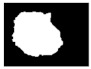	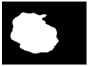	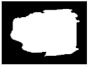	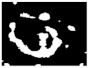	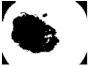	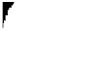
**PH2_IMD211**	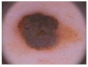	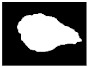	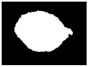	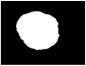	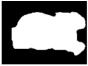	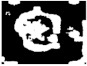	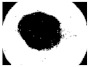	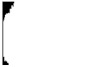
**PH2_IMD284**	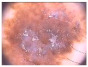	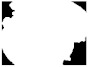	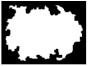	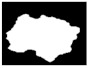	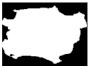	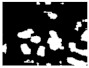	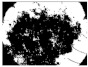	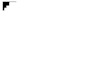
**PH2_IMD285**	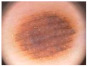	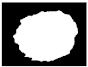	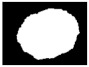	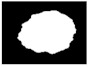	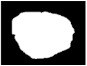	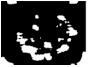	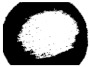	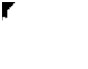
**PH2_IMD348**	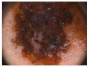	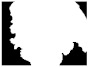	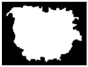	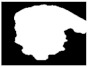	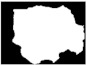	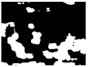	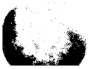	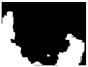
**PH2_IMD349**	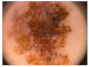	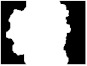	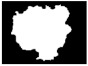	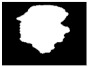	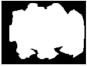	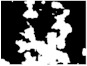	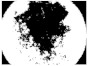	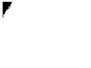
**PH2_IMD403**	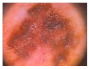	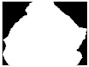	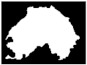	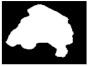	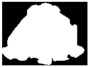	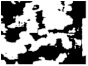	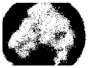	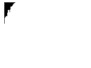
**PH2_IMD404**	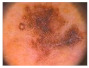	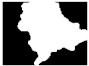	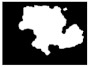	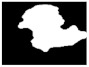	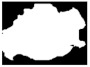	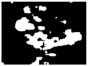	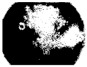	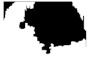
**PH2_IMD407**	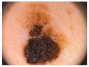	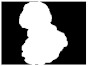	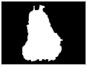	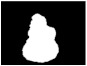	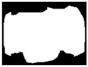	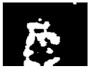	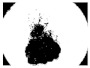	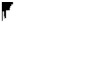
**PH2_IMD409**	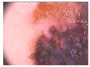	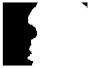	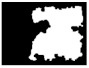	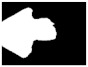	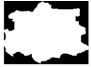	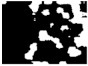	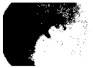	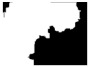
**PH2_IMD425**	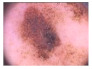	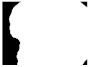	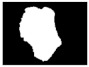	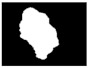	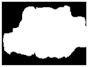	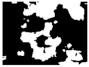	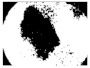	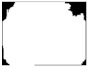
**PH2_IMD426**	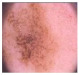	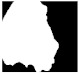	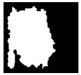	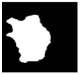	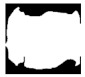	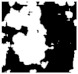	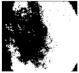	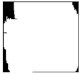
**PH2_IMD435**	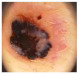	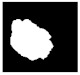	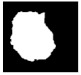	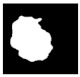	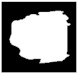	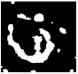	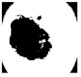	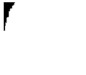

**Table 6:** Quantitative evaluation on a subset of ISIC dataset (benign).

Methods	Med-Sensitivity (%)	Med-Specificity (%)	Med-Accuracy (%)	Med-Jaccard-Index (Ji)	Med-Dice-Coefficient (Dc)
CV_LS [67]	75.26	82.29	79.88	0.48	0.65
MORPH_CV_LS [10]	76.92	**100.00**	93.68	0.75	0.85
MORPH_GAC_LS [10]	93.66	0.51	61.63	0.40	0.55
ADAPTIVE_THRESH [68]	**97.95**	0.67	25.39	0.25	0.39
ISODATA_THRESH [69]	24.07	0.22	7.33	0.06	0.11
MEAN_THRESH [70]	6.00	7.25	9.08	0.02	0.03
TRIANGLE_THRESH [71]	9.53	1.89	4.82	0.02	0.05
OTSU_THRESH [72]	23.72	0.23	7.32	0.06	0.11
SALIENCY_MAP [55]	75.37	89.94	81.10	0.48	0.65
SRM_OBJ [53]	79.05	99.96	93.04	0.73	0.84
EGBS_OBJ [52]	80.61	99.62	83.91	0.43	0.61
SLIC_CLUST [66]	19.70	0.35	6.76	0.05	0.10
GASISUK (Proposed)	80.68	**100.00**	**95.17**	**0.80**	**0.89**

**Table 7:** Quantitative evaluation on a subset of PH2 dataset (benign).

Methods	Med-Sensitivity (%)	Med-Specificity (%)	Med-Accuracy (%)	Med-Jaccard-Index (Ji)	Med-Dice-Coefficient (Dc)
CV_LS [67]	14.00	53.66	47.16	0.04	0.08
MORPH_CV_LS [10]	77.49	**99.70**	93.40	0.72	0.84
MORPH_GAC_LS [10]	95.61	42.50	51.91	0.28	0.44
ADAPTIVE_THRESH [68]	91.13	3.91	21.22	0.18	0.31
ISODATA_THRESH [69]	27.28	11.24	14.85	0.06	0.12
MEAN_THRESH [70]	7.94	20.67	19.43	0.02	0.04
TRIANGLE_THRESH [71]	16.88	13.50	14.83	0.04	0.08
OTSU_THRESH [72]	26.57	11.29	14.76	0.06	0.12
SALIENCY_MAP [55]	66.96	85.62	77.66	0.36	0.53
SRM_OBJ [53]	**100.00**	3.45	23.43	0.21	0.34
EGBS_OBJ [52]	89.48	75.01	50.95	0.15	0.26
SLIC_CLUST [66]	20.94	15.94	17.13	0.07	0.13
GASISUK (Proposed)	89.42	99.55	**96.76**	**0.85**	**0.92**

**Table 8:** Quantitative evaluation on ISIC dataset (malignant).

Methods	Med-Sensitivity (%)	Med-Specificity (%)	Med-Accuracy (%)	Med-Jaccard Index (Ji)	Med-Dice-Coefficient (Dc)
CV_LS [67]	60.67	79.87	73.36	0.40	0.57
MORPH_CV_LS [10]	73.78	**100.00**	91.27	0.72	0.84
MORPH_GAC_LS [10]	93.23	54.89	67.68	0.48	0.65
ADAPTIVE_THRESH [68]	**96.85**	0.10	31.27	0.31	0.47
ISODATA_THRESH [69]	28.04	1.73	11.73	0.09	0.16
MEAN_THRESH [70]	13.25	12.29	14.36	0.05	0.09
TRIANGLE_THRESH [71]	20.40	2.37	9.12	0.06	0.11
OTSU_THRESH [72]	27.44	1.87	11.66	0.08	0.16
SALIENCY_MAP [55]	62.97	92.12	77.95	0.46	0.63
SRM_OBJ [53]	84.22	99.66	87.92	0.64	0.78
EGBS_OBJ [52]	78.23	99.34	81.52	0.48	0.65
SLIC_CLUST [66]	24.62	2.25	11.50	0.08	0.15
GASISUK (Proposed)	80.02	99.97	**93.45**	**0.78**	**0.88**

**Table 9:** Quantitative evaluation on PH2 dataset (malignant).

Methods	Med-Sensitivity (%)	Med-Specificity (%)	Med-Accuracy (%)	Med-Jaccard Index (Ji)	Med-Dice-Coefficient (Dc)
CV_LS [67]	26.22	45.06	34.55	0.21	0.34
MORPH_CV_LS [10]	53.86	**100.00**	71.74	0.54	0.70
MORPH_GAC_LS [10]	81.67	83.20	74.87	0.68	0.81
ADAPTIVE_THRESH [68]	92.70	3.72	62.59	0.62	0.76
ISODATA_THRESH [69]	24.93	21.22	23.72	0.18	0.30
MEAN_THRESH [70]	28.31	20.72	25.69	0.18	0.31
TRIANGLE_THRESH [71]	21.59	23.01	21.31	0.14	0.24
OTSU_THRESH [72]	23.07	21.22	23.16	0.17	0.29
SALIENCY_MAP [55]	36.16	87.15	59.08	0.32	0.49
SRM_OBJ [53]	**99.66**	10.82	57.50	0.55	0.71
EGBS_OBJ [52]	8.89	96.52	36.46	0.09	0.16
SLIC_CLUST [66]	49.10	22.22	43.14	0.40	0.57
GASISUK (Proposed)	70.39	99.50	**80.94**	**0.70**	**0.82**

**Table 10:** Illustrative analysis of deep learning algorithms on ISIC 2017 testing dataset segmentation task.

Methods	Sensitivity (%)	Specificity (%)	Accuracy (%)	Jaccard Index (Ji)	Dice-Coefficient (Dc)
Bi et al. [21]	86.20	96.71	94.08	0.78	0.86
Sarker et al. [22]	81.60	98.30	93.60	0.78	**0.88**
Liu et al. [23]	88.76	96.51	94.32	0.79	0.87
Al-mansi et al. [24]	85.40	96.69	94.03	0.77	0.87
Phan et al. [25]			**94.55**	**0.80**	**0.88**
Abhishek et al. [73]	87.06	95.16	92.20	0.76	0.84
Yuan et al. [26]	82.50	97.50	93.40	0.77	0.85
Shan et al. [27]	83.82	**98.65**	93.71	0.76	0.85
Tang et al. [28]	89.53	96.32	94.31	0.79	0.87

## Data Availability

Publicly available ISIC datasets used in this study can be found at https://www.isic-archive.com/#!/topWithHeader/onlyHeaderTop/gallery?filter=%5B%5D (accessed on 29 July 2021). Similarly, publicly available PH2 datasets used can be found at https://www.dropbox.com/s/k88qukc20ljnbuo/PH2Dataset.rar (accessed on 29 July 2021). Description of datasets used in this study can also be found at https://github.com/dokuboyejo/gasisuk-pub (accessed on 29 July 2021).
